# Case Report: Massive sustained-release valproate overdose with prolonged gastric tablet retention and partial pharmacobezoar formation managed by endoscopic removal and delayed extracorporeal therapy

**DOI:** 10.3389/fphar.2026.1923807

**Published:** 2026-09-17

**Authors:** Pochen Li, Yang Wu, Qianping Zhang, Danxia Ge, Yujiao Li, Fangyu Yu

**Affiliations:** 1 Intensive Care Unit, Ningbo Municipal Hospital of Traditional Chinese Medicine (TCM), Affiliated Hospital of Zhejiang Chinese Medical University, Ningbo, China; 2 Department of Respiratory, Ningbo Municipal Hospital of Traditional Chinese Medicine (TCM), Affiliated Hospital of Zhejiang Chinese Medical University, Ningbo, China

**Keywords:** case report, endoscopic tablet removal, pharmacobezoar, sustained-release formulation, valproate overdose

## Abstract

**Background:**

Valproate poisoning is a potentially life-threatening condition that may result in central nervous system depression, hyperammonemia, metabolic disturbances, and multiorgan dysfunction. Management of massive sustained-release valproate overdose remains challenging because delayed gastrointestinal absorption may lead to prolonged toxicity despite conventional decontamination measures.

**Case Presentation:**

A 23-year-old woman with a history of previous suicide attempts was admitted approximately 6 hours after ingesting ninety 500-mg sustained-release valproate tablets (total dose 45 g) with alcohol. Upon arrival, she exhibited severe agitation and delirium. Endotracheal intubation was performed for airway protection because she was unable to cooperate with treatment and was at risk of aspiration. Gastric lavage and cathartic therapy were subsequently administered. The initial serum valproate concentration was 1,093.66 μg/mL. Because incomplete decontamination was suspected, bedside upper gastrointestinal endoscopy was performed approximately 11.5 h after ingestion. Fourteen intact and markedly swollen tablets were removed; some were separate, whereas others were adherent and partially aggregated, supporting partial pharmacobezoar formation and prolonged gastric drug retention. During intensive care unit management, the patient developed severe hyperammonemia (peak ammonia 205 μmol/L), hyperlactatemia with mixed metabolic acidosis and respiratory alkalosis, acute kidney injury, marked creatine kinase elevation, hypernatremia, and suspected transient central diabetes insipidus. Meropenem was administered empirically from May 31 to June 3 because impaired airway defenses after intubation raised concern for aspiration-related infection, although no definite infectious focus or causative pathogen was identified. Because of substantial financial hardship, extracorporeal treatment was initially deferred. It was initiated at 20:00 on June 1, approximately 62 h after ingestion and 56 h after hospital admission. Two consecutive 2-h sessions of hemoperfusion combined with CVVH were followed by CVVH alone until 08:30 on June 2. The serum valproate concentration decreased from 397.05 μg/mL before extracorporeal treatment to 12.72 μg/mL during ongoing CVVH and was 8.61 μg/mL approximately 70 h after treatment cessation. The patient was discharged on June 15 without evident neurological, cognitive, or motor impairment.

**Conclusion:**

Massive sustained-release valproate overdose may cause prolonged gastric retention of intact and partially aggregated tablets, thereby sustaining systemic toxicity. In selected patients, endoscopic tablet removal may provide toxicological source control, while extracorporeal treatment may remain beneficial even when its initiation is delayed. The respective contributions of endoscopy, meropenem exposure, spontaneous elimination, and extracorporeal treatment cannot be separated in this single case.

## 1 Introduction

Valproic acid (VPA) is a widely prescribed antiepileptic and mood-stabilizing agent. Acute VPA overdose may result in central nervous system depression, respiratory failure, hyperammonemia, metabolic disturbances, hepatotoxicity, and, in severe cases, multiorgan dysfunction (Sztajnkrycer, 2002). Although most patients can be managed with supportive care, massive overdose involving sustained-release formulations presents unique challenges because delayed absorption and prolonged drug release may result in persistent toxicity despite conventional gastrointestinal decontamination (Bialer et al., 1985).

Pharmacobezoar formation has increasingly been recognized as an important mechanism contributing to delayed drug absorption in massive sustained-release drug overdoses (Alrazooqi et al., 2025). Aggregated tablets retained within the gastrointestinal tract may function as a continuous drug reservoir, leading to prolonged systemic exposure, delayed peak serum concentrations, and recurrent or persistent toxicity (Gosselin et al., 2025). A recent report described a 30-cm clumped valproate pharmacobezoar associated with delayed neuromuscular toxicity (Alrazooqi et al., 2025). Therefore, the novelty of the present case does not lie in being the first report of a valproate pharmacobezoar, but in documenting endoscopic separation and quantitative retrieval of retained tablets after prior gastric lavage, followed by a precisely characterized delayed extracorporeal treatment course.

Current management strategies for severe VPA intoxication include gastrointestinal decontamination, supportive care, correction of metabolic complications, and extracorporeal treatment when indicated (Liu et al., 2023). Furthermore, in resource-limited settings, patients may not have timely access to extracorporeal treatment, which poses additional challenges for clinical management (Bernasconi et al., 2026).

We report a case of massive sustained-release valproate overdose with an initial serum valproate concentration exceeding 1,000 μg/mL. Endoscopy performed approximately 11.5 h after ingestion demonstrated 14 retained, intact, and markedly swollen tablets, some of which were adherent and partially aggregated. The separate tablets were removed individually with a stone-retrieval basket, whereas the adherent tablets were separated before retrieval. Despite delayed extracorporeal treatment, the patient recovered without evident neurological, cognitive, or motor impairment at discharge. This case highlights therapeutic endoscopic source control after incomplete gastric lavage, serial toxicokinetic monitoring, and individualized extracorporeal treatment when intermittent hemodialysis is not immediately available.

## 2 Case presentation

A 23-year-old woman with a history of previous suicide attempts was brought to the emergency department approximately 6 hours after intentionally ingesting ninety 500-mg sustained-release sodium valproate tablets (total estimated dose 45 g) together with alcohol. According to family members, the ingestion occurred at approximately 06:00 on May 30, 2026. She was found at 11:30 and arrived at the emergency department at 12:06.

Upon presentation, the patient was in a state of marked psychomotor agitation and delirium. Initial vital signs were as follows: blood pressure 126/69 mmHg, heart rate 85 beats/min, respiratory rate 19 breaths/min, and oxygen saturation within the normal range on room air. Neurological examination revealed agitation and confusion without focal neurological deficits; the pupils were equal and reactive to light. Because of persistent severe agitation and delirium, inability to cooperate with treatment, aspiration risk, concerns regarding airway protection, and anticipated progression of severe intoxication, endotracheal intubation was performed at 12:40. The indication for intubation was airway protection rather than established respiratory failure. Gastric lavage and cathartic therapy were subsequently initiated.

The patient was admitted to the intensive care unit (ICU) shortly thereafter. On ICU admission, her blood pressure was 109/63 mmHg, heart rate 70 beats/min, and respiratory rate 22 breaths/min under mechanical ventilation. The serum valproate concentration measured at 14:50 on May 30 was 1,093.66 μg/mL, exceeding the upper therapeutic range by more than tenfold. At 07:56 on May 31, serum creatinine was 68 μmol/L, creatine kinase (CK) was 150 U/L, and serum albumin was 43.0 g/L. Free valproate concentrations were not measured because this assay was unavailable at our institution.

Despite gastric lavage, ongoing gastrointestinal absorption was suspected because of the massive ingestion of a sustained-release formulation. Bedside upper gastrointestinal endoscopy was therefore performed at 17:30 on the day of admission, approximately 11.5 h after ingestion (Figure 1). Fourteen intact but markedly swollen sustained-release sodium valproate tablets were identified in the stomach. Some tablets remained separate, whereas others were adherent and partially aggregated into a pharmacobezoar-like mass. Separate tablets were retrieved individually using a stone-retrieval basket; the adherent tablets were first separated endoscopically and then removed. These findings demonstrate prolonged gastric retention and partial aggregation rather than a single large consolidated bezoar.

**FIGURE 1:**
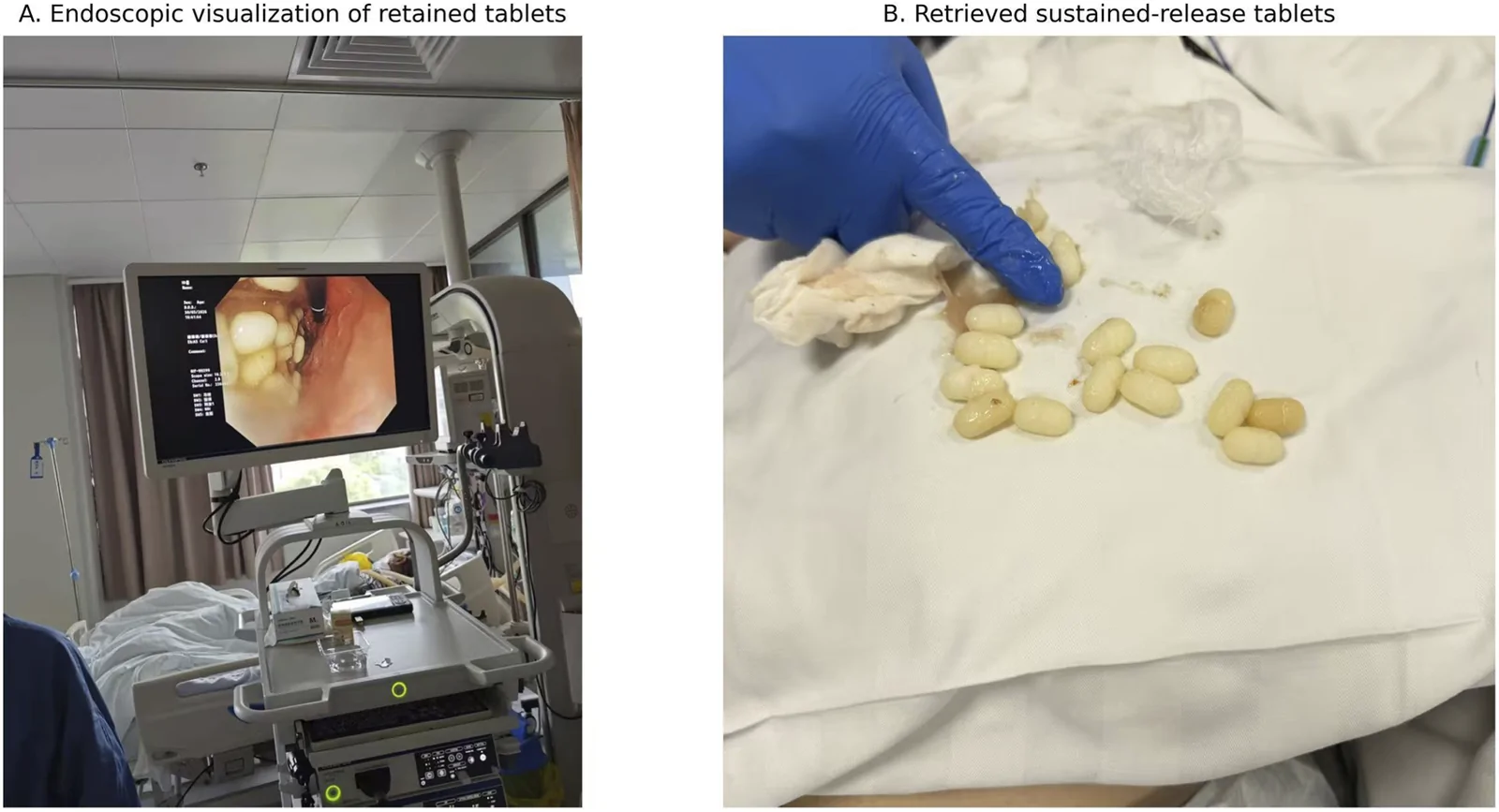
Endoscopic findings demonstrating prolonged gastric retention and partial pharmacobezoar formation after massive sustained-release valproate ingestion. **(A)** Upper gastrointestinal endoscopy approximately 11.5 h after ingestion demonstrated multiple intact and markedly swollen tablets; some tablets remained separate, whereas others were adherent and partially aggregated. **(B)** Fourteen intact and swollen sustained-release valproate tablets after endoscopic retrieval. Separate tablets were removed individually with a stone-retrieval basket; adherent tablets were first separated and then retrieved. Panel B documents tablet morphology after removal but does not alone demonstrate the *in situ* aggregation pattern.

The first arterial blood gas obtained at 16:53 on May 30 while the patient was receiving an FiO2 of 40% showed pH 7.42, PaCO2 30 mmHg, actual bicarbonate 19.5 mmol/L, base excess −4.1 mmol/L, lactate 7.7 mmol/L, and an anion gap of 18 mmol/L. These findings indicated hyperlactatemic metabolic acidosis with concurrent respiratory alkalosis, rather than isolated respiratory alkalosis. Lactate decreased to 5.6 mmol/L at 20:15, 4.2 mmol/L at 23:42, 3.8 mmol/L at 05:42 on May 31, and 1.5 mmol/L by 15:59 on June 1. At 20:29 on May 30, plasma ammonia was markedly elevated at 205 μmol/L. Intravenous L-ornithine L-aspartate was administered at 10 g twice daily, lactulose therapy was initiated, and deep sedation was maintained with midazolam and remifentanil.

During the first hospital day, the patient developed progressive polyuria and hypernatremia. Total urine output reached approximately 5,100 mL during the initial 16-h observation period. Serum sodium increased to 158 mmol/L, measured serum osmolality was 330 mOsm/kg, and urine sodium was 112 mmol/L. Transient central diabetes insipidus was suspected. Continuous vasopressin infusion was administered for approximately 3 hours (total dose 6 units), and desmopressin 3.56 μg was administered intravenously at 09:22 on May 31. Urine output and serum sodium subsequently improved without recurrence after treatment cessation. However, urine osmolality and formal confirmatory testing were not performed; therefore, central diabetes insipidus remained a tentative diagnosis. Follow-up brain computed tomography on June 2 revealed no significant intracranial abnormality.

Meropenem (1 g every 8 h) was initiated empirically at 09:23 on May 31 because impaired airway defenses after intubation raised concern for aspiration-related infection. No definite infectious focus or causative pathogen was identified. Meropenem was discontinued at 08:59 on June 3 after clinical reassessment and improvement in the inflammatory profile.

Although extracorporeal treatment was recommended because of the extremely elevated serum valproate concentration, severe hyperammonemia, and evolving systemic toxicity, the patient’s family initially declined hemoperfusion and continuous renal replacement therapy because of substantial financial hardship. Early management therefore consisted of gastrointestinal source control, supportive care, ammonia-lowering therapy, sedation, and close laboratory monitoring while the clinical team continued to discuss the need for extracorporeal treatment with the family.

Serial measurements showed that serum valproate decreased from 1,093.66 μg/mL at 14:50 on May 30–594.05 μg/mL at 16:00 on May 31 and 397.05 μg/mL at 11:30 on June 1. Plasma ammonia decreased from 205 μmol/L at 20:29 on May 30–198 μmol/L at 01:43 on May 31, 178 μmol/L at 15:47 on May 31, and 150 μmol/L at 10:39 on June 1. Serum creatinine increased from 68 μmol/L at 07:56 on May 31–106 μmol/L at 07:41 on June 1. CK increased from 150 U/L to 770 U/L over the same interval and subsequently peaked at 2512 U/L at 08:49 on June 2. Serum albumin decreased from 43.0 g/L on May 31–36.8 g/L on June 1 and 31.8 g/L at 08:49 on June 2.

After repeated discussions, the family consented to extracorporeal treatment. Treatment was initiated at 20:00 on June 1, approximately 62 h after ingestion and approximately 56 h after hospital admission. The first KCM-150 hemoperfusion cartridge was used with CVVH from 20:00 to 22:00, followed immediately by a second cartridge from 22:00 to 00:00. After completion of the two hemoperfusion sessions, CVVH alone was continued from 00:00 to 08:30 on June 2. Hemoperfusion was performed at a blood flow rate of 100 mL/min. During CVVH, blood flow was 150 mL/min, prescribed effluent dose was approximately 35 mL/kg/h, systemic heparin anticoagulation was used, and the filtration fraction was maintained at 25%–29%. Intermittent hemodialysis was not available emergently at our institution. The selected strategy reflected local availability and team experience and aimed to rapidly adsorb circulating valproate, continuously clear free drug and ammonia, treat acute kidney injury, and mitigate redistribution or rebound after hemoperfusion.

At 05:51 on June 2, while CVVH was still ongoing, serum valproate had decreased to 12.72 μg/mL. CVVH was discontinued at 08:30. Plasma ammonia normalized to 11 μmol/L at 10:35, approximately 2 hours after treatment cessation. At 08:49, serum creatinine was 48 μmol/L, CK was 2512 U/L, and albumin was 31.8 g/L. A follow-up valproate concentration obtained at 06:30 on June 5, approximately 70 h after CVVH discontinuation, was 8.61 μg/mL, showing no evidence of a sustained late rebound at that time point. Because serial concentrations were not obtained during the early post-treatment period, a transient early rebound could not be completely excluded. CK subsequently decreased to 1986 U/L on June 3 and 1024 U/L on June 4, while creatinine decreased to 42 and 40 μmol/L, respectively (Figure 2).

**FIGURE 2:**
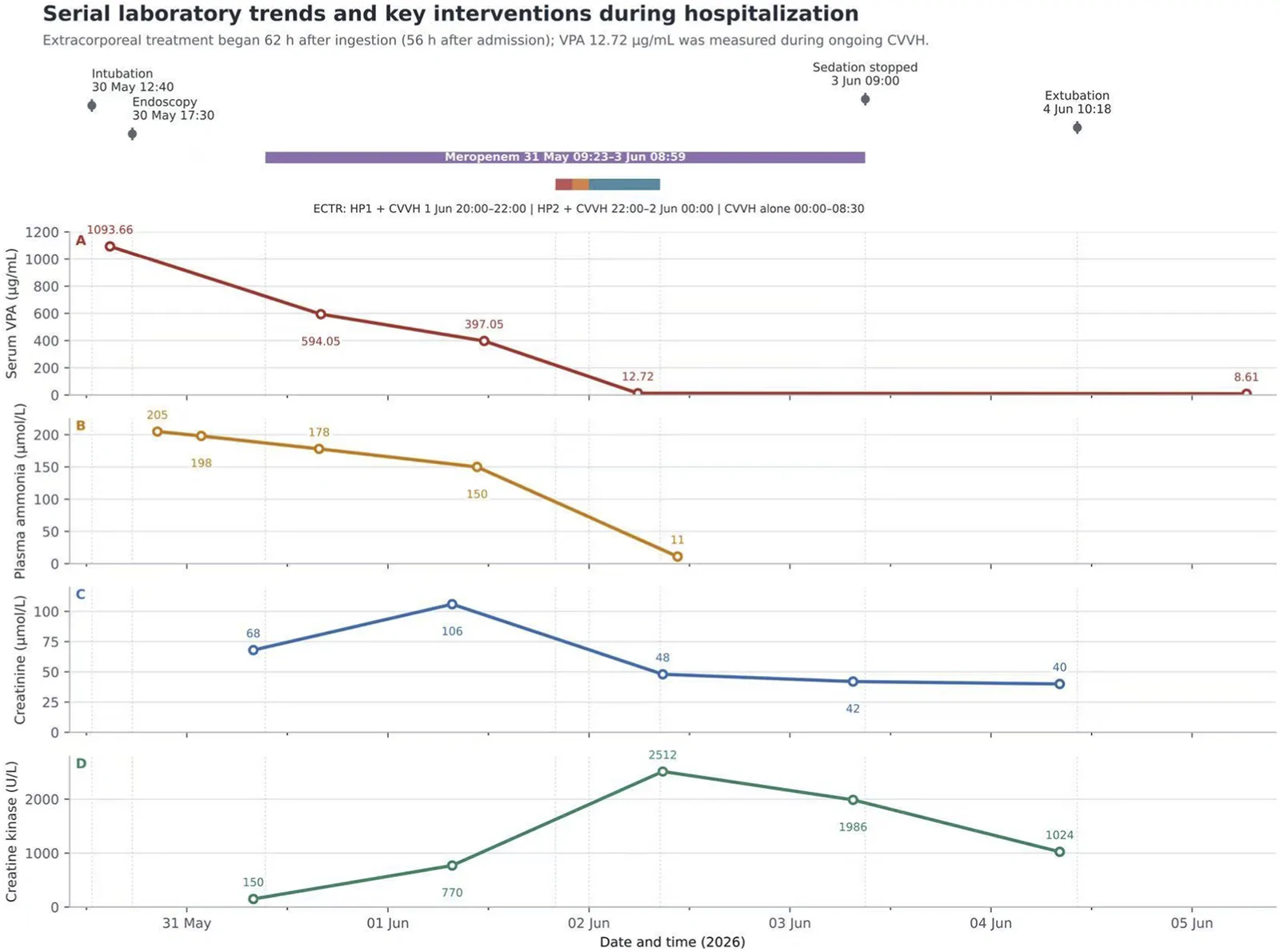
Serial laboratory trends and key clinical interventions during hospitalization. The upper timeline shows intubation, endoscopic tablet removal, meropenem exposure, two consecutive hemoperfusion sessions combined with CVVH, subsequent CVVH alone, discontinuation of sedation, and extubation. Panels **(A–D)** show serum valproate, plasma ammonia, serum creatinine, and CK, respectively. Extracorporeal treatment began at 20:00 on June 1, 62 h after ingestion and approximately 56 h after admission. The valproate concentration of 12.72 μg/mL was obtained during ongoing CVVH; the follow-up value of 8.61 μg/mL was obtained approximately 70 h after CVVH cessation.

Sedative medications were discontinued at 09:00 on June 3. The patient regained consciousness without focal neurological deficits. No overt convulsive activity was observed; however, bedside electroencephalography was unavailable at our institution, and non-convulsive seizures could not be completely excluded. She was extubated at 10:18 on June 4 and transferred from the ICU to a general ward on June 5. She was discharged on June 15 without evident neurological, cognitive, or motor impairment. Her family was advised to monitor the risk of recurrent self-harm, and psychiatric and psychological follow-up was recommended. No post-discharge follow-up data were available.

## 3 Discussion

### 3.1 Pharmacobezoar formation and the potential role of endoscopic tablet removal

One of the most noteworthy features of this case was the persistence of multiple intact sustained-release valproate tablets within the stomach despite prior gastric lavage. Approximately 11.5 h after ingestion, endoscopy revealed 14 markedly swollen but structurally preserved tablets. Some remained separate, whereas others were adherent and partially aggregated. Thus, the endoscopic findings support prolonged gastric tablet retention and partial pharmacobezoar formation, but not a single large consolidated mass. Their direct endoscopic separation and retrieval reduced a plausible ongoing source of absorption.

Pharmacobezoars are aggregates of medications that accumulate within the gastrointestinal tract and function as a continuous reservoir for drug absorption. They have been described following overdose of sustained-release, enteric-coated, and poorly soluble formulations. Several mechanisms may contribute to pharmacobezoar formation, including ingestion of large numbers of tablets, delayed gastric emptying, reduced gastrointestinal motility, anticholinergic effects, altered consciousness, and alcohol co-ingestion. In massive overdoses, tablets may adhere to one another and undergo progressive swelling, thereby delaying dissolution and prolonging systemic exposure (Alrazooqi et al., 2025; Wang et al., 2025).

In addition, a descriptive analysis of 632 pharmacobezoar reports in VigiBase found that 432 (68.4%) were classified as serious; among 158 reports associated with acute intoxication, 156 (98.7%) were serious. By encompassing a wide range of drugs, this study provides broader pharmacovigilance context for the clinical relevance and heterogeneity of pharmacobezoar formation. However, because the analysis was based on spontaneous reports involving multiple drug classes, it cannot establish incidence, causality, or a valproate-specific risk (Carollo et al., 2025).

Sustained-release valproate preparations appear particularly susceptible to this phenomenon. Compared with immediate-release formulations, sustained-release tablets exhibit slower dissolution kinetics and prolonged gastrointestinal transit. Consequently, serum concentrations may continue to rise or remain elevated for extended periods after ingestion. Previous reports have described delayed peak concentrations occurring many hours after overdose, occasionally resulting in clinical deterioration despite apparently appropriate initial management (Yousef et al., 2025). Therefore, conventional decontamination measures alone may be insufficient in selected patients with massive sustained-release valproate intoxication.

An *in vitro* model of extended-release divalproex sodium demonstrated formation of a solid 5 × 6-cm pharmacobezoar after 2 hours that remained stable for 24 h (López et al., 2026). In the present case, 14 intact and markedly swollen tablets persisted in the stomach approximately 11.5 h after ingestion despite prior gastric lavage. Some tablets were adherent and partially aggregated, whereas others remained separate. These findings are consistent with delayed dissolution and a persistent gastrointestinal drug reservoir, although the morphology differed from the previously reported 30-cm consolidated valproate pharmacobezoar (Alrazooqi et al., 2025).

The present case demonstrates several findings consistent with ongoing gastrointestinal drug sequestration. First, the patient ingested approximately 45 g of sustained-release sodium valproate. Second, 14 intact tablets remained in the stomach more than 11 h after ingestion despite gastric lavage. Third, serum valproate remained markedly elevated over the following day. However, the concentration declined after several overlapping interventions, including endoscopic removal, spontaneous elimination, meropenem exposure, and subsequent extracorporeal treatment; therefore, the contribution of any single intervention cannot be quantified.

Current toxicology guidelines emphasize gastrointestinal decontamination using gastric lavage and activated charcoal in selected circumstances; however, evidence regarding endoscopic removal of retained drug material remains limited. Endoscopic intervention is not routinely recommended for most poisonings because of procedural risks and uncertain benefit. Nevertheless, in carefully selected patients with suspected pharmacobezoar formation, direct visualization and removal of retained tablets may provide a unique opportunity to eliminate an ongoing source of toxicity. In the present case, endoscopy was not performed as a routine procedure but rather because persistent gastric retention was suspected following massive ingestion of a sustained-release formulation. The successful retrieval of multiple intact tablets supports the clinical rationale for this approach.

Although the precise contribution of endoscopic tablet removal cannot be quantified, the clinical significance of this intervention may extend beyond simple gastrointestinal decontamination. In cases of pharmacobezoar formation, retained tablets function as a persistent toxic reservoir capable of sustaining systemic drug exposure long after ingestion. Therefore, endoscopic removal may be viewed as a form of toxicological source control, analogous to source control strategies employed in critical care and infectious diseases. The findings of this case underscore the importance of identifying persistent gastrointestinal drug reservoirs in patients with unexpectedly prolonged toxicity. Endoscopic source control may serve as a valuable adjunct to conventional supportive and extracorporeal therapies when pharmacobezoar formation is suspected.

### 3.2 Delayed extracorporeal therapy and the value of staged toxicological management

Extracorporeal treatment is widely recognized as an effective therapeutic modality in severe valproate intoxication because valproate exhibits relatively favorable physicochemical characteristics for extracorporeal removal at toxic concentrations (Raděj et al., 2019). The EXTRIP Workgroup recommends extracorporeal treatment in patients with markedly elevated serum valproate concentrations or severe clinical manifestations, including coma, respiratory failure requiring mechanical ventilation, shock, cerebral edema, or significant hyperammonemia (Ghannoum et al., 2015).

The patient fulfilled accepted indications for extracorporeal treatment. Her serum valproate concentration was 1,093.66 μg/mL, exceeding the EXTRIP threshold at which extracorporeal treatment is suggested, and she developed severe hyperammonemia and evolving multiorgan toxicity (Ghannoum et al., 2015).

Valproate is highly but saturably bound to albumin. Serum albumin was 43.0 g/L early in the course, decreased to 36.8 g/L on June 1, and reached 31.8 g/L on June 2. Although albumin was initially normal, the extreme valproate concentration would be expected to saturate available binding sites, while the subsequent decline in albumin may have further increased the unbound fraction. Consequently, total valproate concentrations may not have fully reflected pharmacologically active exposure. At the same time, an increased unbound fraction would make more valproate accessible to extracorporeal removal. Because unbound valproate was not measured, the magnitude and timing of these effects cannot be quantified.

Extracorporeal treatment was initially deferred because of substantial financial hardship. During this period, management relied on endoscopic source control, supportive care, ammonia-lowering therapy, mechanical ventilation, deep sedation, and close monitoring. Serum valproate gradually decreased, but severe hyperammonemia and evolving organ dysfunction persisted, supporting escalation to extracorporeal treatment once consent was obtained.

Extracorporeal treatment began at 20:00 on June 1. Two consecutive 2-h sessions of hemoperfusion combined with CVVH were followed by 8.5 h of CVVH alone. The selected approach reflected local availability and team experience. Hemoperfusion was used for rapid adsorption, whereas continued CVVH was intended to support ongoing clearance of free valproate and ammonia, treat acute kidney injury, and reduce redistribution or rebound risk. A valproate concentration of 12.72 μg/mL was measured during ongoing CVVH, and the concentration was 8.61 μg/mL approximately 70 h after treatment cessation. No sustained late rebound was detected at this time point; however, an early transient rebound could not be excluded because serial valproate measurements immediately after extracorporeal treatment were unavailable.

Importantly, the present case should not be interpreted as evidence that delayed extracorporeal treatment is equivalent to early intervention. Rather, it illustrates that clinically meaningful benefits may still be achieved when extracorporeal therapy cannot be initiated immediately because of logistical, financial, or patient-related barriers. In many regions, particularly in resource-limited settings, delays in accessing hemoperfusion or continuous renal replacement therapy remain common. Under such circumstances, implementation of a staged toxicological management strategy may provide an effective bridge until definitive toxin removal becomes feasible.

Another notable observation is that successful recovery was achieved through the combined effect of multiple interventions rather than any single therapeutic modality. Endoscopic removal of retained tablets likely reduced ongoing gastrointestinal absorption, supportive measures mitigated secondary organ injury, and extracorporeal treatment facilitated rapid toxin clearance once initiated. This sequence highlights the importance of viewing severe poisoning as a dynamic process requiring continuous reassessment and escalation of therapy according to evolving clinical conditions.

Therefore, although early extracorporeal treatment remains the preferred approach for severe valproate intoxication, delayed initiation should not be regarded as futile. In selected patients, particularly when substantial systemic toxicity persists despite conservative treatment, extracorporeal therapy may still provide significant clinical benefit and contribute to favorable outcomes.

### 3.3 Meropenem–valproate interaction as a pharmacokinetic confounder

Carbapenems can rapidly and substantially reduce serum valproate concentrations by interrupting valproate-glucuronide hydrolysis and enterohepatic recirculation; concurrent use is generally avoided in patients receiving valproate for seizure control (Hernández-Ramos et al., 2021; Zhang et al., 2024; Suzuki et al., 2011; Torii et al., 2002). In this case, meropenem was administered empirically because impaired airway defenses after intubation raised concern for aspiration-related infection, rather than for the purpose of enhancing valproate elimination. No definite infectious focus or causative pathogen was identified, and meropenem was discontinued after clinical reassessment. Although reports have described reduced valproate concentrations after carbapenem exposure in overdose (Juurlink, 2025; Muñoz-Pichuante et al., 2020; Thomas et al., 2020), the decline observed here also followed gastric lavage, endoscopic tablet removal, spontaneous elimination, and extracorporeal treatment. Meropenem should therefore be regarded only as a pharmacokinetic confounder, not as an established treatment for valproate poisoning.

### 3.4 Hyperammonemia and systemic toxic manifestations of massive valproate intoxication

In addition to prolonged gastrointestinal absorption and delayed toxin clearance, the patient developed severe hyperammonemia, hyperlactatemia with mixed metabolic acidosis and respiratory alkalosis, acute kidney injury, marked CK elevation, hypernatremia, and transient polyuria raising suspicion of central diabetes insipidus. These abnormalities likely reflected the combined effects of extreme valproate exposure and secondary metabolic disturbances.

Among these findings, hyperammonemia represented one of the most clinically significant complications. Plasma ammonia concentrations reached 205 μmol/L despite the absence of substantial hepatocellular injury, with only mild transient elevation of aspartate aminotransferase observed during hospitalization. This pattern is consistent with the well-recognized phenomenon of valproate-induced hyperammonemia, which may occur independently of overt hepatic failure. Several mechanisms have been proposed, including inhibition of mitochondrial β-oxidation, depletion of carnitine stores, impairment of carbamoyl phosphate synthetase activity, and disruption of the urea cycle. These metabolic alterations ultimately reduce ammonia clearance and promote systemic accumulation (Riboldi et al., 2025; Patel and Nagalli, 2024; Jawień et al., 2017).

Interestingly, the neurological manifestations observed in this patient differed from the classical presentation of hyperammonemic encephalopathy. Rather than progressive somnolence or coma, the patient initially exhibited marked agitation, delirium, and excessive respiratory drive associated with persistent respiratory alkalosis. Although altered mental status is common in severe valproate poisoning, the spectrum of neuropsychiatric manifestations may be broader than traditionally appreciated. It is possible that hyperammonemia contributed not only to neurological dysfunction but also to abnormal central respiratory regulation in this case.

The patient developed transient acute kidney injury, with serum creatinine increasing from 68 to 106 μmol/L before recovering during extracorporeal treatment. CK increased from 150 U/L to a peak of 2512 U/L despite ongoing sedation and analgesia at the time of the peak. No overt convulsive activity was observed. The CK elevation was considered multifactorial, with possible contributions from severe agitation and increased muscular activity during the early phase, metabolic disturbances, and systemic valproate toxicity. However, because bedside EEG was unavailable, non-convulsive seizure activity could not be completely excluded.

Another unusual finding was transient polyuria, hypernatremia, and hyperosmolality. Urine output reached approximately 5,100 mL over 16 h, serum sodium increased to 158 mmol/L, and serum osmolality was 330 mOsm/kg. The abnormalities improved after short-term vasopressin infusion and a single intravenous dose of desmopressin. However, urine osmolality and formal confirmatory testing were not performed. Consequently, central diabetes insipidus remained a tentative diagnosis rather than a confirmed complication. Whether the polyuria was directly related to valproate toxicity, hyperammonemia, or another transient physiological disturbance remains uncertain.

Taken together, these observations underscore the multisystem nature of severe valproate intoxication. Beyond central nervous system depression alone, massive overdose may result in complex metabolic, renal, muscular, and neuroendocrine abnormalities. Early recognition of these complications and prompt supportive management remain essential components of successful treatment.

L-carnitine has been proposed as a therapeutic option in severe valproate intoxication, particularly in patients with marked hyperammonemia, hepatotoxicity, or altered mental status (Rezaee and Abbasgholizadeh, 2026; Gziut and Thanacoody, 2025). The proposed benefits include restoration of mitochondrial β-oxidation, enhancement of ammonia metabolism, and reduction of toxic valproate metabolites. In the present case, L-carnitine was not administered because it was not routinely available at our institution during the treatment period. Nevertheless, normalization of ammonia levels and clinical recovery were achieved through a combination of ammonia-lowering therapy, supportive care, and extracorporeal treatment.

## 4 Conclusion

Massive sustained-release valproate overdose may be complicated by prolonged gastric retention and partial aggregation of intact swollen tablets, sustaining gastrointestinal absorption despite conventional decontamination. In this case, endoscopy approximately 11.5 h after ingestion demonstrated both separate and adherent tablets; endoscopic separation and retrieval provided direct toxicological source control.

This case does not represent the first endoscopically documented valproate pharmacobezoar. Its distinct contribution is the quantitative endoscopic retrieval of 14 retained tablets after gastric lavage, including separation of partially aggregated tablets, together with a precisely reconstructed delayed extracorporeal treatment course. Meaningful recovery was achieved through multiple overlapping interventions, but their individual effects cannot be separated in a single case. The absence of free valproate measurement, early post-treatment concentration monitoring, bedside EEG, confirmatory testing for central diabetes insipidus, and long-term follow-up should be considered when interpreting the findings.

Recognition of prolonged gastric tablet retention, selective endoscopic source control, serial toxicokinetic monitoring, and individualized use of available extracorporeal modalities may be important in severe sustained-release valproate intoxication.

## Data Availability

The original contributions presented in the study are included in the article/Supplementary Material, further inquiries can be directed to the corresponding author.
