# Supplementary material for: Case Report: Massive sustained-release valproate overdose with prolonged gastric tablet retention and partial pharmacobezoar formation managed by endoscopic removal and delayed extracorporeal therapy
Source: Front Pharmacol. 2026 Sep 17;17:1923807. doi: 10.3389/fphar.2026.1923807 (PMC13626886; doi:10.3389/fphar.2026.1923807)
Supplement: Supplementary file 1 [file Table1.docx]

**CARE Checklist**

*Massive Sustained-Release Valproate Overdose With Prolonged Gastric Tablet Retention and Partial Pharmacobezoar Formation Managed by Endoscopic Removal and Delayed Extracorporeal Therapy*

| **Topic** | **Item** | **CARE checklist item** | **Reported on** | **Status** |
| --- | --- | --- | --- | --- |
| **Title** | 1 | The diagnosis or intervention of primary focus followed by the words ‘case report’. | Title | Completed |
| **Keywords** | 2 | Two to five keywords that identify the diagnoses or interventions in this case report, including ‘case report’. | Keywords | Completed |
| **Abstract** | 3a | Introduction: What is unique about this case and what does it add to the medical literature? | Abstract | Completed |
| **Abstract** | 3b | Main symptoms and/or important clinical findings. | Abstract | Completed |
| **Abstract** | 3c | The main diagnoses, therapeutic interventions, and outcomes. | Abstract | Completed |
| **Abstract** | 3d | Conclusion: What are the main ‘take-away’ lessons from this case? | Abstract | Completed |
| **Introduction** | 4 | One or two paragraphs summarizing why this case is unique; may include references. | Introduction | Completed |
| **Patient information** | 5a | De-identified demographic and other patient-specific information. | Case Presentation | Completed |
| **Patient information** | 5b | Main concerns and symptoms of the patient. | Case Presentation | Completed |
| **Patient information** | 5c | Medical, family, psychosocial, and genetic history, including relevant past interventions and their outcomes. | Case Presentation | Completed |
| **Patient information** | 5d | Relevant past interventions and their outcomes. | Not applicable | Not applicable |
| **Clinical findings** | 6 | Relevant physical examination and other significant clinical findings. | Case Presentation | Completed |
| **Timeline** | 7 | Important dates and times in the case, organized as a timeline. | Figure 2 | Completed |
| **Diagnostic assessment** | 8a | Diagnostic methods, including physical examination, laboratory testing, imaging, and surveys. | Case Presentation | Completed |
| **Diagnostic assessment** | 8b | Diagnostic challenges, such as access, financial, cultural, or language-related factors. | Case Presentation | Completed |
| **Diagnostic assessment** | 8c | Diagnosis, including other diagnoses considered. | Case Presentation | Completed |
| **Diagnostic assessment** | 8d | Prognostic characteristics, when applicable. | Not applicable | Not applicable |
| **Therapeutic intervention** | 9a | Types of therapeutic intervention, such as pharmacologic, surgical, preventive, or self-care interventions. | Case Presentation | Completed |
| **Therapeutic intervention** | 9b | Administration of interventions, including dosage, strength, and duration. | Case Presentation | Completed |
| **Therapeutic intervention** | 9c | Changes in interventions, with rationale. | Case Presentation | Completed |
| **Follow-up and outcomes** | 10a | Clinician- and patient-assessed outcomes, when available. | Case Presentation | Completed |
| **Follow-up and outcomes** | 10b | Important follow-up diagnostic and other test results. | Case Presentation | Completed |
| **Follow-up and outcomes** | 10c | Intervention adherence and tolerability, including how these were assessed. | Not applicable | Not applicable |
| **Follow-up and outcomes** | 10d | Adverse and unanticipated events. | Not applicable | Not applicable |
| **Discussion** | 11a | Strengths and limitations of the approach to this case. | Discussion | Completed |
| **Discussion** | 11b | Relevant medical literature. | References | Completed |
| **Discussion** | 11c | The rationale for conclusions, including assessment of possible causes. | Discussion | Completed |
| **Discussion** | 11d | The primary ‘take-away’ lessons of the case report. | Discussion | Completed |
| **Patient perspective** | 12 | The patient should share their perspective or experience, when possible. | Not available | Not available |
| **Informed consent** | 13 | Did the patient provide informed consent? | Ethics Statement | Yes, Completed |
